# Self-inflicted thoracoabdominal nail-gun injury presenting as STEMI: diagnostic challenges in a time-critical care pathway

**DOI:** 10.1093/jscr/rjag914

**Published:** 2026-10-10

**Authors:** Charles Belling, Hye Rim Suh, Kayna Fichadia, Saimurooban Muralidaran, Arooj Ahmed, Susmit Roy, Jeremy Hsu

**Affiliations:** Department of General Surgery, Westmead Hospital, Cnr Hawkesbury Road and Darcy Rd, Westmead, New South Wales 2145, Australia; Westmead Trauma Service, Westmead Hospital, Cnr Hawkesbury Road and Darcy Rd, Westmead, New South Wales 2145, Australia; Department of General Surgery, Westmead Hospital, Cnr Hawkesbury Road and Darcy Rd, Westmead, New South Wales 2145, Australia; Western Sydney University, School of Medicine, Locked Bag 1797, Penrith 2751, New South Wales 2751, Australia; Department of Cardiothoracic Surgery, Westmead Hospital, Cnr Hawkesbury Road and Darcy Rd, Westmead, New South Wales 2145, Australia; Westmead Trauma Service, Westmead Hospital, Cnr Hawkesbury Road and Darcy Rd, Westmead, New South Wales 2145, Australia; Department of General Surgery, Westmead Hospital, Cnr Hawkesbury Road and Darcy Rd, Westmead, New South Wales 2145, Australia; Westmead Trauma Service, Westmead Hospital, Cnr Hawkesbury Road and Darcy Rd, Westmead, New South Wales 2145, Australia; Department of General Surgery, Westmead Hospital, Cnr Hawkesbury Road and Darcy Rd, Westmead, New South Wales 2145, Australia; Westmead Trauma Service, Westmead Hospital, Cnr Hawkesbury Road and Darcy Rd, Westmead, New South Wales 2145, Australia

**Keywords:** penetrating cardiac injury, nail-gun injury, thoracoabdominal trauma, ST-elevation myocardial infarction, delayed diagnosis, situation awareness, diagnostic reassessment

## Abstract

A 60-year-old male with a history of depression and a prior nail-gun thoracic injury was found in shock at home, with inferior ST-segment elevation on the paramedic electrocardiogram prompting pre-hospital ST-elevation myocardial infarction pathway activation. Initial high-sensitivity troponin was elevated, consistent with the presumed diagnosis. Normal coronary angiography and preserved ventricular function were discordant with the patient’s ongoing haemodynamic instability. Four small anterior chest puncture wounds and radiopaque foreign bodies demonstrated on chest radiograph and angiographic imaging did not initially prompt consideration of penetrating injury. Computed tomography pulmonary angiography, performed to exclude pulmonary embolism, identified four nail-gun projectiles penetrating the heart and stomach. The patient underwent sternotomy and laparotomy with repair of cardiac, diaphragmatic, and gastric injuries. The case illustrates the challenges of situation awareness and diagnostic reassessment within time-critical, protocol-driven care.

## Introduction

Penetrating trauma accounts for <5% of trauma presentations in Australia [1], and cardiac nail-gun injuries are rare, confined to isolated case reports [2, 3]. Nail-gun injuries are associated with greater survival to hospital presentation because of lower projectile velocity, reduced cavitation injury, and tamponade physiology limiting exsanguination [4]. Self-inflicted nail-gun injuries are associated with increased intensive care unit (ICU) admission and mortality compared with accidental injuries [5]. Electrocardiographic abnormalities including ST-elevation may occur after cardiac trauma in the absence of coronary occlusion [6]. Prior reports have described left ventricular nail-gun injuries presenting with chest pain, troponin elevation, and normal coronary angiography [7]. This case highlights the challenges of diagnostic reassessment during time-critical, protocol-driven care.

## Case report

A 60-year-old male with depression and a prior nail-gun chest injury (2018) was found in shock by paramedics after family discovered the patient collapsed. A timeline of key events and their diagnostic significance is presented in Table 1. Prehospital observations revealed diaphoresis, vomiting, left shoulder pain, and shock (initial: BP 40/palp, HR 90 regular, SpO2 90% room air). Penetrating wounds were not documented in the paramedic assessment, and the nail-gun injury was not disclosed. Inferior ST-elevation on paramedic electrocardiogram (ECG) prompted ST-elevation myocardial infarction (STEMI) pathway activation. Four ‘small anterior chest lacerations’ were noted by emergency staff. Initial high-sensitivity troponin T was elevated at 483 ng/l (reference <14 ng/l). Dual antiplatelet loading was administered.

**Table 1:** Clinical timeline of presentation, investigation, and diagnosis

	Approximate time (24 h)	Clinical findings and events
**Day 1**	15:00	In shock Self-inflicted mechanism not disclosed
	15:30	Inferior ST-elevation on paramedic ECG Pre-hospital STEMI pathway activated
	16:01	Triaged Remains shocked requiring adrenaline aliquots
	16:05	ED resuscitation assessment Four ‘small anterior chest lacerations’ noted High-sensitivity troponin T 483 ng/l Dual antiplatelet loading
	16:20	Emergency coronary angiography—no culprit lesion Preserved LV function on echocardiography Four nails overlying the cardiac silhouette visible on angiographic imaging
	17:19	Angiogram report finalized: no culprit lesion reported
	17:30–20:00	Post-angiography period: Dried blood over anterior chest attributed to shaving during catheter laboratory preparation ‘Diagnostic dilemma’ recognized - APO and PE considered CXR demonstrated bilateral infiltrates attributed to pulmonary oedema or aspiration and a single radiopaque nail CTPA ordered but delayed by system constraints ABG demonstrated type 2 respiratory failure
	20:00–21:00	ICU admission - Intubated and vasopressor-dependent Four punctate anterior chest wounds with subcutaneous emphysema documented Next of kin reported discovery of a suicide note
**Day 2**	00:26	CXR to confirm central line placement demonstrates multiple nails projected over the left lower chest
	02:37	CTPA (to exclude PE) incidentally demonstrates four nail-gun projectiles penetrating the heart and stomach
	03:00	Referral to trauma and cardiothoracic surgery Four cutaneous wounds within the cardiac box correlated with projectile trajectories on imaging
	05:00	Theatre (~14 h after paramedic retrieval) Sternotomy and cardiopulmonary bypass - retrieval three intracardiac nails - repair of ventricular defects and diaphragm laparotomy - retrieval one abdominal nail - repair gastric injuries, splenectomy

ABG, arterial blood gas; APO, acute pulmonary oedema; CTPA, CT pulmonary angiography; CXR, chest radiograph; ECG, electrocardiogram; ED, emergency department; ICU, intensive care unit; LV, left ventricular; PE, pulmonary embolism; STEMI, ST-elevation myocardial infarction.

The patient was intubated for emergency coronary angiography which demonstrated no culprit lesion. Four nails overlying the cardiac silhouette were visible on angiographic images but were not identified as projectiles (Fig. 1). Preserved left ventricular function was confirmed on echocardiography. A post-angiography chest radiograph was obtained to evaluate the differential diagnosis of pulmonary oedema and incidentally captured a single radiopaque nail at the film periphery (Fig. 2a).

**Figure 1:**
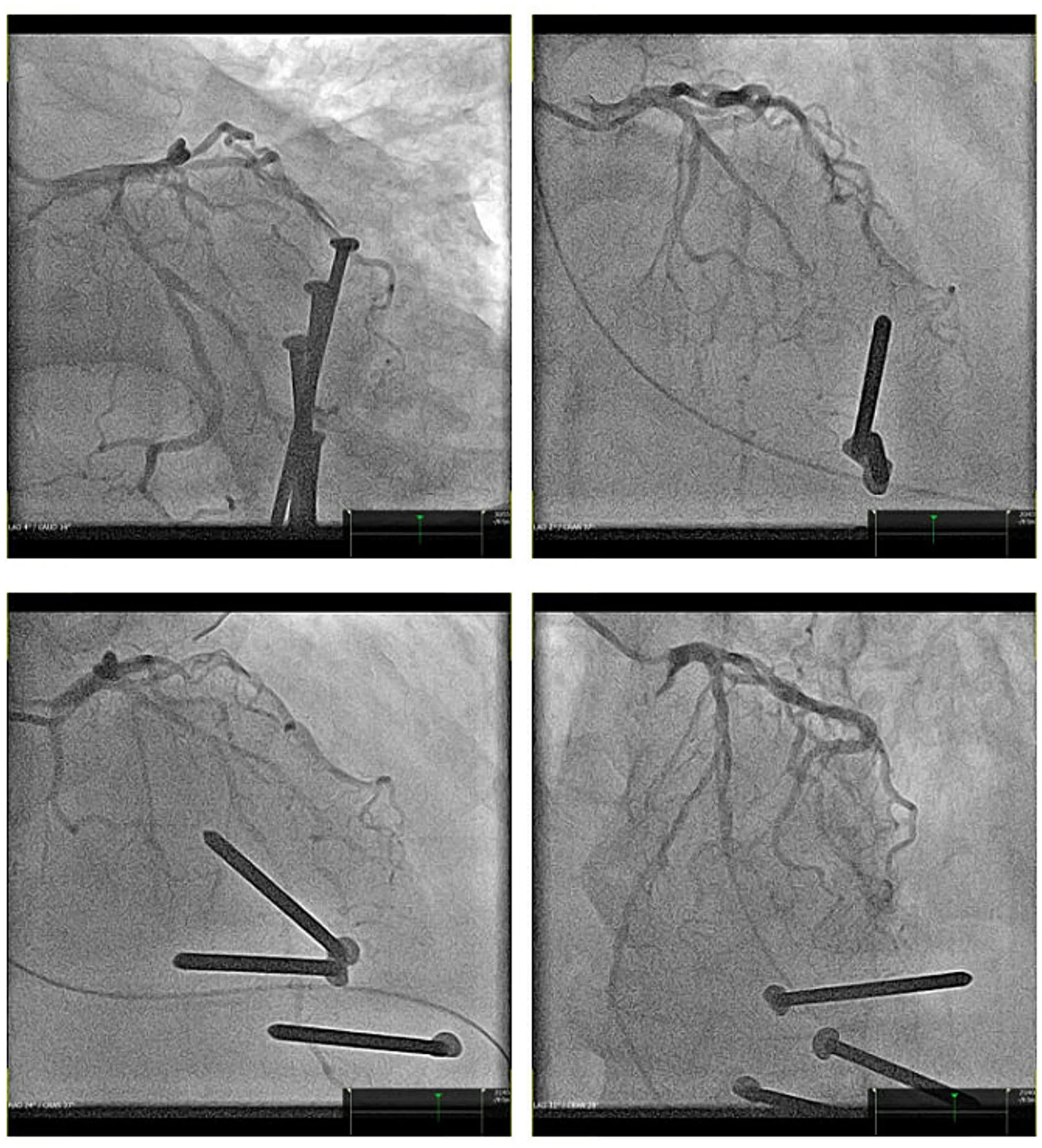
Fluoroscopic images in composite from coronary angiography demonstrating radiopaque nails overlying the cardiac silhouette.

**Figure 2:**
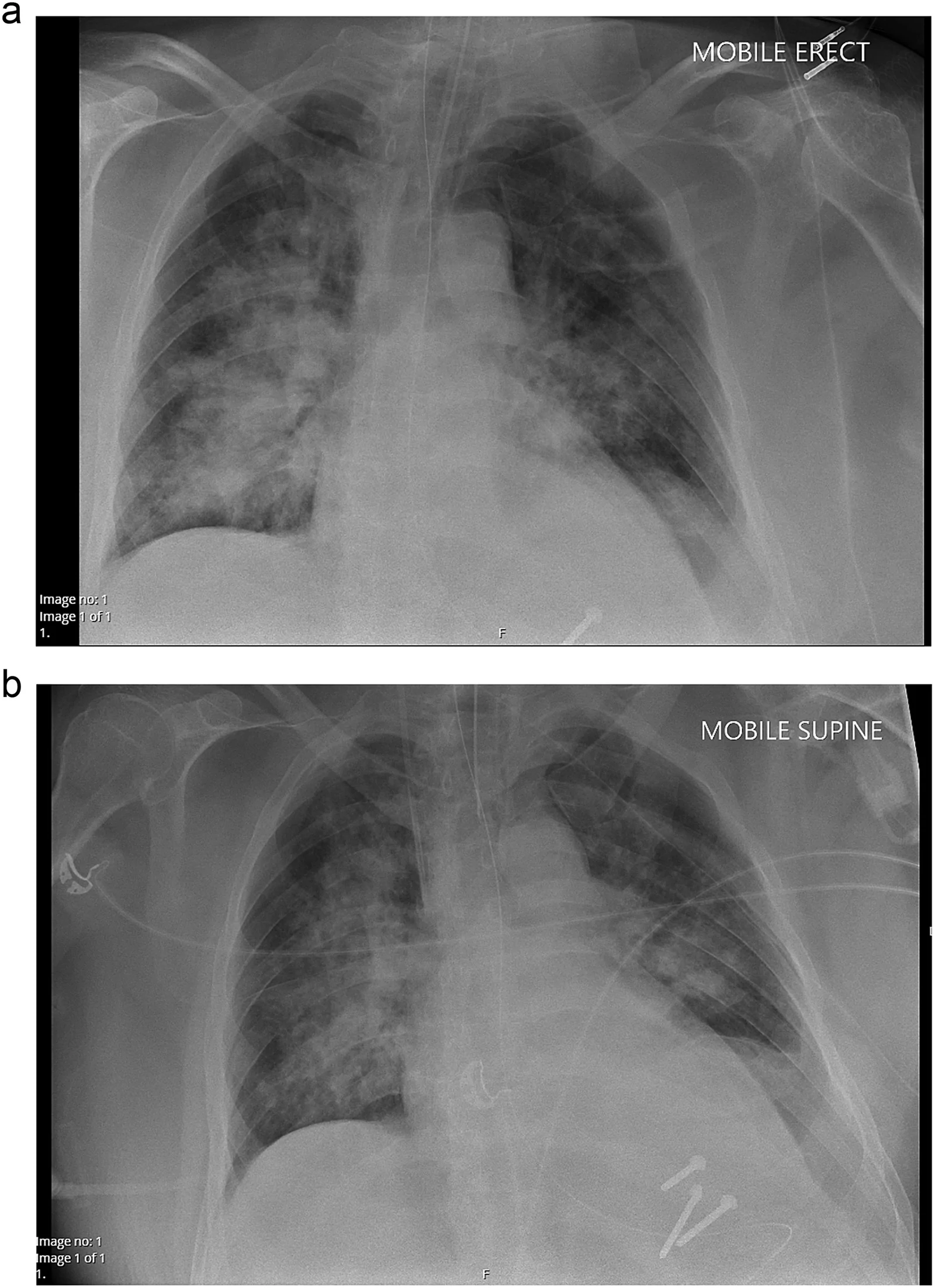
Sequential chest radiographs both obtained post-angiography; (a) mobile supine chest radiograph obtained to evaluate for acute pulmonary oedema; a partially imaged radiopaque foreign body is present at the film periphery; (b) mobile supine chest radiograph to confirm central line placement demonstrating multiple radiopaque foreign bodies projecting over the lower thorax and upper abdomen.

Following normal coronary angiography, the case was recognized as a ‘diagnostic dilemma’. Pulmonary embolism (PE) was raised as a differential diagnosis between the anaesthetic and interventional cardiology teams, and computed tomography pulmonary angiography (CTPA) was ordered, though delayed by system constraints.

A second chest radiograph demonstrated bilateral infiltrates, attributed to pulmonary oedema or aspiration. Radiopaque foreign bodies were again captured projecting over the lower thorax and upper abdomen (Fig. 2b).

Intubated and vasopressor dependent, the patient was transferred to the ICU. Admission notes contained a description of four punctate anterior chest wounds with subcutaneous emphysema. At the time of ICU admission, collateral history from the next of kin revealed discovery of a suicide note. A CTPA performed the following morning to exclude PE incidentally demonstrated four nail-gun projectiles penetrating the heart and stomach (Fig. 3).

**Figure 3:**
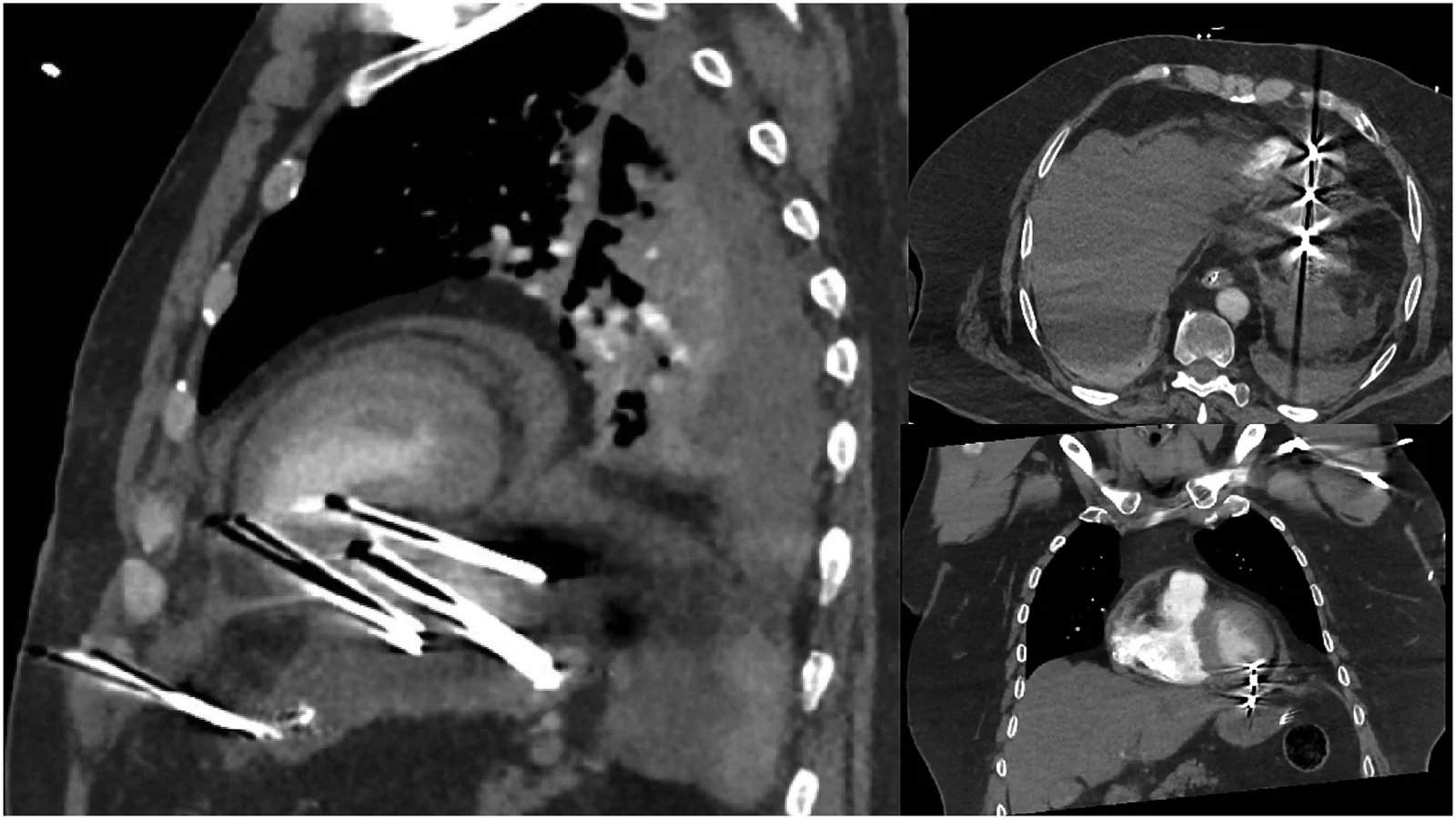
Composite CT imaging (axial, coronal, and sagittal reconstructions) demonstrating nail-gun projectiles penetrating the inferior left ventricle with trajectories extending towards the stomach; associated findings included haemopericardium, left haemopneumothorax, haemoperitoneum, and bilateral pulmonary consolidation.

Following a review of the CTPA, four cutaneous wounds within the cardiac box were recognized to correspond to the projectile trajectories identified on imaging (Fig. 4).

**Figure 4:**
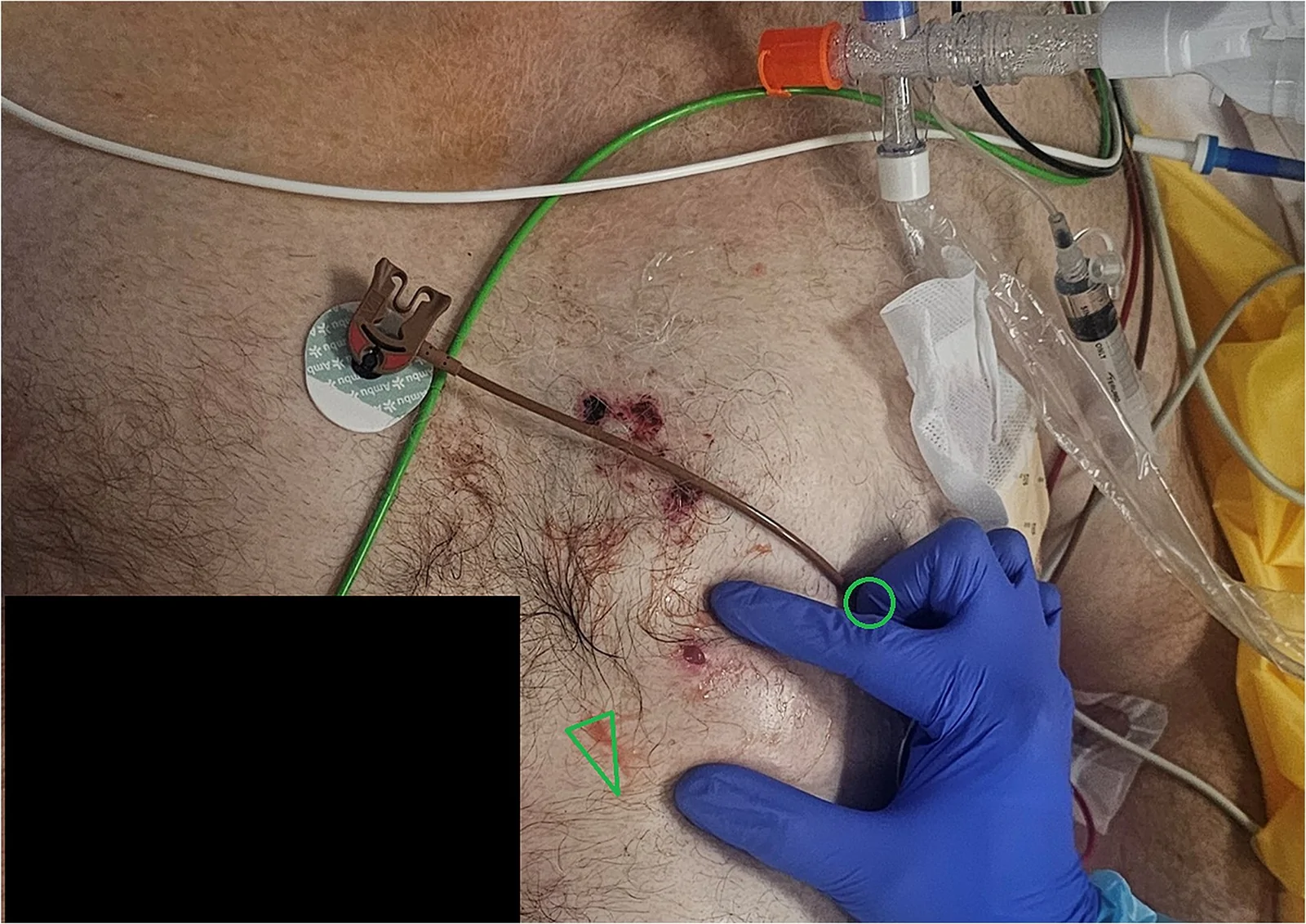
Four puncture wounds located within the cardiac box; triangle and circle mark level of xiphisternum and left nipple.

The patient was then transferred to theatre, arriving ~14 h after paramedic retrieval.

Median sternotomy was performed and cardiopulmonary bypass was established. Four cardiac defects were identified at the apex and infero-lateral left ventricle, with three nails retrieved from the pericardium and heart (Fig. 5). The cardiac defects were repaired with four pledgeted 3–0 sutures (Fig. 6) and the associated left hemidiaphragm punctures with 4–0 Prolene.

**Figure 5:**
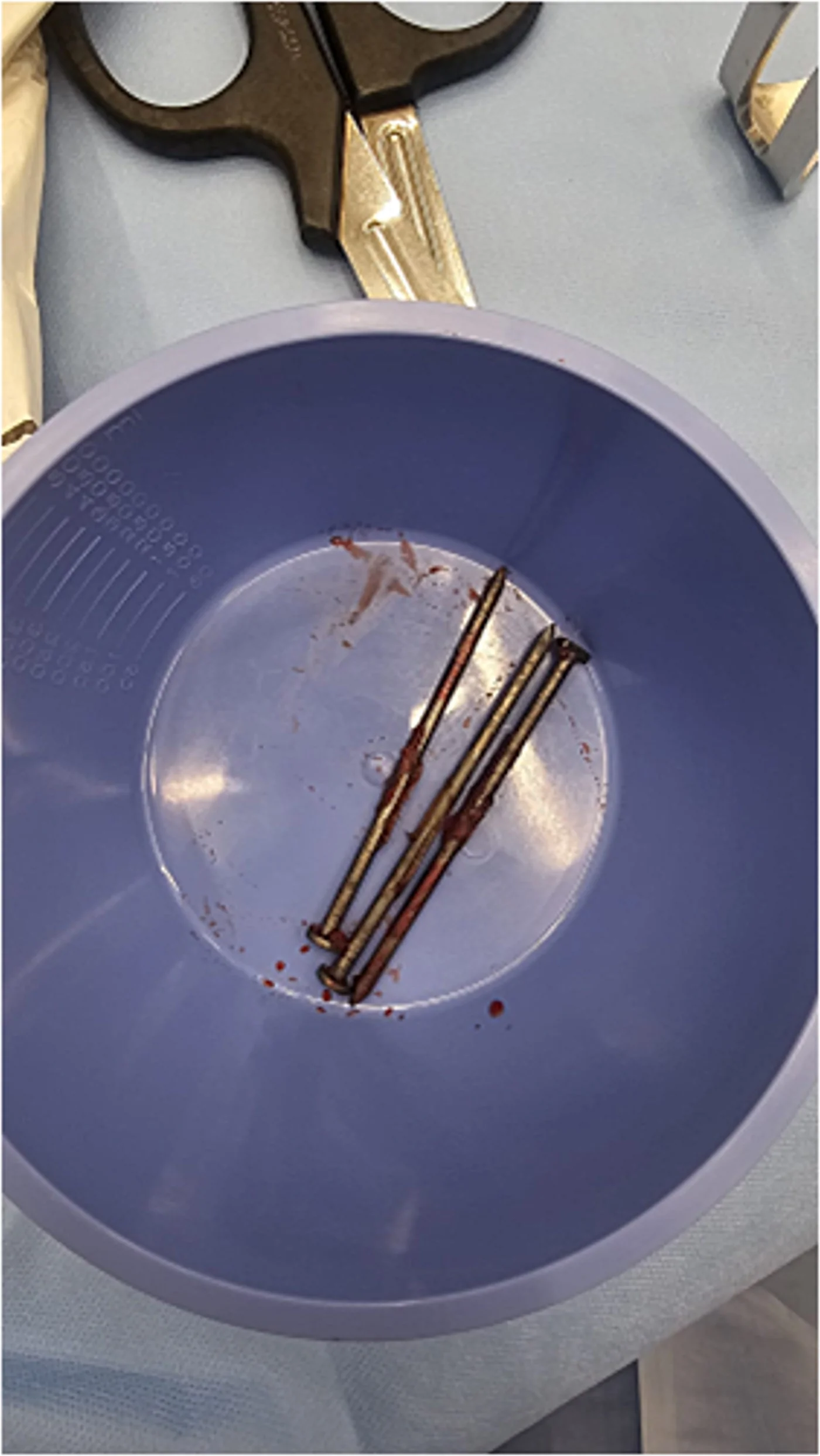
Three nails retrieved at sternotomy from the pericardium and heart; a fourth nail (not pictured) was retrieved separately at laparotomy.

**Figure 6:**
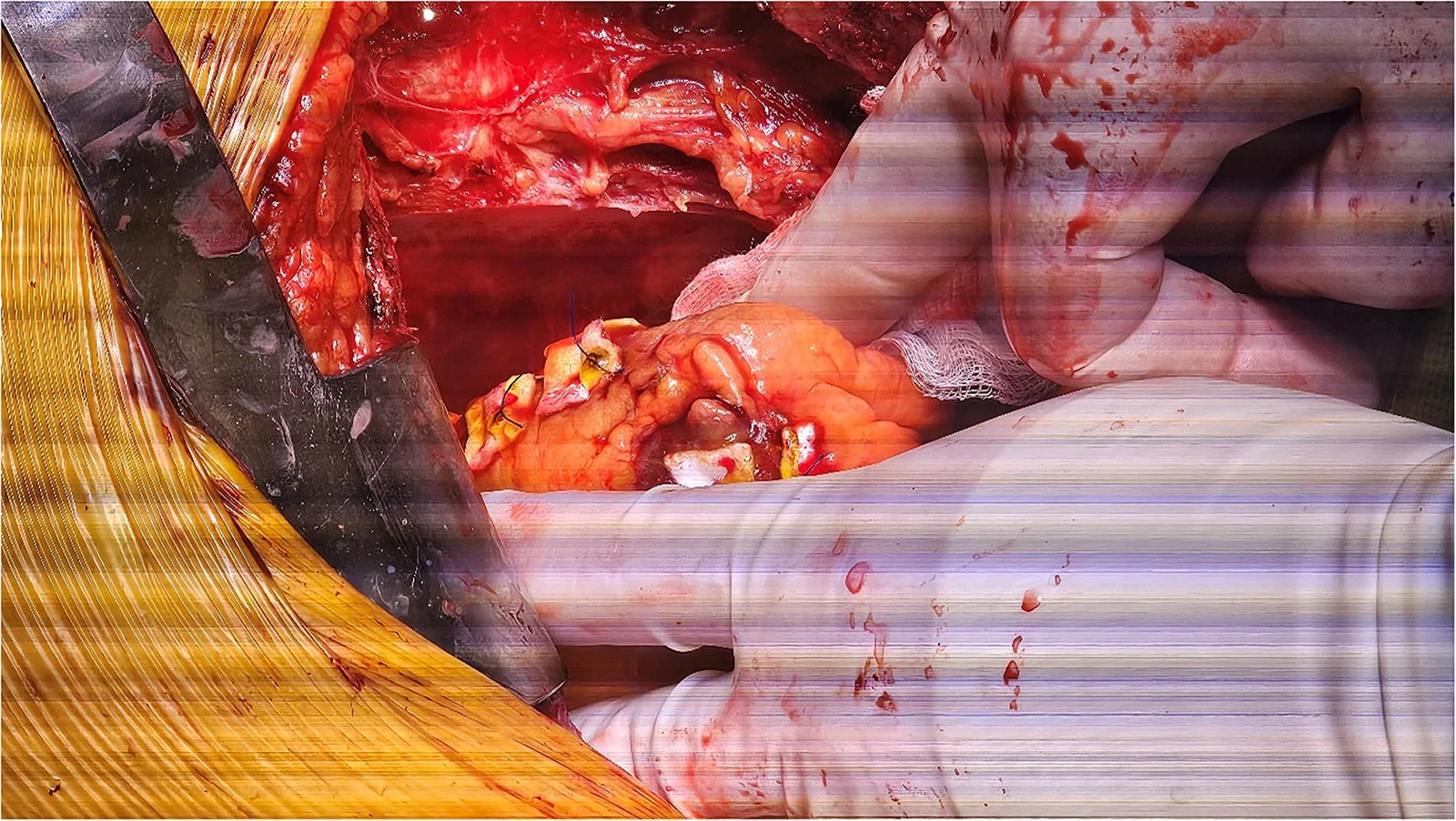
Completed pledgeted repair of left ventricular injuries using 3–0 Prolene sutures.

Laparotomy demonstrated the fourth, inferiormost nail impacted between the lower left ribs, its trajectory associated with gastric injury. The nail was removed in retrograde fashion, and two anterior gastric puncture wounds were repaired primarily. Left upper quadrant haemorrhage attributed to splenic hilar injury necessitated splenectomy. Relook laparotomy on Day 2 demonstrated satisfactory haemostasis, and methylene blue testing confirmed integrity of the gastric repair. The patient was extubated on postoperative Day 7, with no neurological deficit, and at the time of writing remained an inpatient undergoing functional and psychiatric recovery.

## Discussion

Time-critical, protocol-driven pathways improve outcomes by compressing the interval to definitive care and reducing variation in care delivery [8, 9]. This presentation ultimately represented unstable penetrating thoracoabdominal trauma requiring urgent operative intervention [6, 8]. However, inferior ST-elevation on ECG prompted activation of a STEMI pathway at first medical contact [9]. The benefits of protocol-driven systems are therefore realized only when the underlying clinical problem has been correctly identified and patients remain on the appropriate pathway as new information emerges [8, 9]. Accurate clinical assessment and ongoing reassessment remain critical throughout the patient’s trajectory of care. As examination findings, imaging and collateral history accumulated, evidence of penetrating thoracoabdominal trauma repeatedly emerged. Yet these findings were repeatedly interpreted within the prevailing medical diagnostic framework, delaying recognition of the underlying surgical diagnosis.

As demonstrated in Table 1, the diagnosis was not delayed by absence of information. Anterior chest wounds had been documented, radiopaque foreign bodies repeatedly captured on imaging, persistent shock despite normal coronary angiography observed, and collateral history suggestive of self-harm obtained before penetrating trauma was recognized. The definitive diagnosis followed reinterpretation of these existing findings after CTPA demonstrated multiple trans-thoracoabdominal projectiles, highlighting that recognition emerged through reframing of existing information rather than acquisition of new information.

The observation that information was present but not initially integrated into a unifying diagnosis can be considered through situation awareness frameworks. The non-technical skills for surgeons framework describes situation awareness as gathering information, understanding its significance and anticipating future states [10, 11]. In this case, many relevant findings were perceived and documented, but their diagnostic implications were not integrated into a coherent understanding of the patient’s underlying condition [10, 11].

Several opportunities for diagnostic reassessment occurred during the patient’s course. However, these findings emerged across multiple teams and clinical handovers during progression through a highly specialized medical pathway. While specialty-specific pathways provide expertise and efficiency, they may also reduce opportunities for alternative disciplinary perspectives.

This case adds to the limited Australian literature describing penetrating cardiac nail-gun injury presenting as acute coronary pathology despite normal coronary angiography [2, 3, 7]. The rarity of this injury pattern may lower clinical suspicion and contribute to diagnostic difficulty [5, 12].

More broadly, this report demonstrates how a surgical problem may progress through a medical pathway despite repeated clinical and radiological evidence of penetrating injury. Protocol-driven systems reduce delays to definitive care, but they remain dependent on accurate identification of the underlying clinical problem [8, 9]. Maintaining situation awareness and integrating examination findings, collateral history, and imaging remain essential to directing patients towards the correct definitive treatment.
